# Simulation model of CA1 pyramidal neurons reveal opposing roles for the Na^+^/Ca^2+^ exchange current and Ca^2+^-activated K^+^ current during spike-timing dependent synaptic plasticity

**DOI:** 10.1371/journal.pone.0230327

**Published:** 2020-03-09

**Authors:** Damien M. O’Halloran

**Affiliations:** Department of Biological Sciences, The George Washington University, Washington DC, United States of America; Universita degli Studi di Napoli Federico II, ITALY

## Abstract

Sodium Calcium exchanger (NCX) proteins utilize the electrochemical gradient of Na^+^ to generate Ca^2+^ efflux (forward mode) or influx (reverse mode). In mammals, there are three unique NCX encoding genes—NCX1, NCX2, and NCX3, that comprise the SLC8A family, and mRNA from all three exchangers is expressed in hippocampal pyramidal cells. Furthermore, mutant *ncx2*^*-*/-^ and *ncx3*^-/-^ mice have each been shown to exhibit altered long-term potentiation (LTP) in the hippocampal CA1 region due to delayed Ca^2+^ clearance after depolarization that alters synaptic transmission. In addition to the role of NCX at the synapse of hippocampal subfields required for LTP, the three NCX isoforms have also been shown to localize to the dendrite of hippocampal pyramidal cells. In the case of NCX1, it has been shown to localize throughout the basal and apical dendrite of CA1 neurons where it helps compartmentalize Ca^2+^ between dendritic shafts and spines. Given the role for NCX and calcium in synaptic plasticity, the capacity of NCX splice-forms to influence backpropagating action potentials has clear consequences for the induction of spike-timing dependent synaptic plasticity (STDP). To explore this, we examined the effect of NCX localization, density, and allosteric activation on forward and back propagating signals and, next employed a STDP paradigm to monitor the effect of NCX on plasticity using back propagating action potentials paired with EPSPs. From our simulation studies we identified a role for the sodium calcium exchange current in normalizing STDP, and demonstrate that NCX is required at the postsynaptic site for this response. We also screened other mechanisms in our model and identified a role for the Ca^2+^ activated K^+^ current at the postsynapse in producing STDP responses. Together, our data reveal opposing roles for the Na^+^/Ca^2+^ exchanger current and the Ca^2+^ activated K^+^ current in setting STDP.

## Introduction

Sodium Calcium exchangers (NCX) are low affinity high capacity (*k*_*cat*_ 2000–5000 s^−1^) antiporters that can rapidly move Ca^2+^ ions across the plasma membrane. NCX can extrude Ca^2+^ at a rate far higher than the plasma membrane Ca^2+^ ATPase pump (*k*_*cat*_ 30–250 s^−1^) [1,2], meaning that NCX is the major regulator of Ca^2+^ homeostasis during periods of elevated intracellular Ca^2+^ concentrations such as vesicle fusion and synaptic transmission. Three NCX genes (SLC8A family–NCX1, NCX2, NCX3) have been cloned and identified in mammals. All three NCX isoforms are expressed in the nervous system at varying levels. NCX1 is considered ubiquitous and therefore expressed in all neurons, although alternative splicing of NCX1 can produce at least 17 splice variants that are selectively expressed in different cell types [3,4]. At the protein level, NCX1 localizes to the presynaptic and postsynaptic sites as well as dendritic shafts and spines [5]. NCX2 does not undergo alternative splicing but is widely expressed throughout the CNS including the medulla, spinal cord, thalamus, cerebellum amygdala, cortex, and hippocampus [4,6,7]. NCX3 is mainly expressed in the nervous system and 5 splice variants have been identified that exhibit tissue and cell specific expression patterns [4,7]. All three NCX isoforms are expressed in the hippocampus where they localize predominately to neuropil, dendrites, and soma [7]. NCX1 is evenly distributed along basal and apical dendrites of CA1 pyramidal cells where it helps compartmentalize Ca^2+^ between dendritic shafts and spines [5]. Furthermore, mutants of *ncx2*^*-*/-^ and *ncx3*^-/-^ exhibit altered learning and memory behavior as well as impaired long-term potentiation (LTP) in the hippocampal CA1 region due to delayed Ca^2+^ clearance after depolarization that alters synaptic transmission [6,8]. In *ncx2*^*-*/-^ mutants the frequency threshold for LTP versus LTD is shifted toward LTP and likely underlies enhancement in hippocampal dependent memory and learning behaviors. Studies on *ncx3*^*-*/-^ mutants revealed a higher basal level of intracellular Ca^2+^ in hippocampal neurons, and field EPSP recordings from CA1 after stimulation of Schaffer collateral fibers demonstrated impaired basal synaptic transmission in *ncx3*^*-*/-^ mutants. NCX3 co-localized with PSD-95, and *ncx3*^*-*/-^ mutants did not display defects in neurotransmitter release, suggesting that NCX3, similarly to NCX2, appears to function primarily at the post-synaptic side of hippocampal subfields required for LTP.

It has been shown that antidromic propagation is known to facilitate associative Hebbian plasticity [9,10]. Hebbian associativity is a function of presynaptic neurotransmitter release and postsynaptic depolarization, and evidence suggests that back-propagating action potentials can provide the necessary postsynaptic depolarization to allow induction of associative synaptic plasticity. In CA1 and cortical layer V pyramidal neurons, these antidromic signaling events significantly enhance the depolarization over what would be observed from synaptic currents alone. The distinct molecular landscape of the dendrite and axon mean that the properties of the back-propagating actions potentials will differ from axonal action potentials in ways that likely reflect their function. Considering the role for NCX and calcium in synaptic plasticity, the capacity of diverse NCX splice-forms to influence depolarization and calcium influx have clear consequences for the induction of synaptic plasticity from antidromic action potentials. Moreover, experimentally teasing apart the effects of NCX in CA1 apical dendrites during antidromic signaling is challenging. Therefore, it seemed of interest to simulate the effects of NCX structure, function, and localization during antidromic propagation and spike-timing dependent synaptic plasticity.

## Materials and methods

### Simulation environment and modeling

All simulations were implemented using NEURON ver. 7.7.1 [11–13] with Python ver. 3.7.0. For orthodromic simulation experiments synaptic stimulation was modeled by positioning 10 glutamatergic synapses randomly along the apical primary dendrite at least 100μm from the soma using the *ExpSyn* class in NEURON with 2ms decay time constant. The *NetCon* network connection class was used to detect spike events at the soma where the threshold value was 10mV (default). Cell discretization was performed using the *d_lambda* rule. Spike half-widths were determined using a HOC *mod* file that included range variables for recording the time (t_1_ in ms) at which the membrane voltage (mV) was greater than threshold (10mV), and the time (t_2_ in ms) at which the membrane voltage was back below threshold; the half-width (ms) is measured by subtracting these two time points (i.e. t_2_ –t_1_). For antidromic simulation experiments, the *iClamp* class was used to create a point process to introduce current at the soma which back-propagates through the apical dendrite. Recording vectors were introduced along the apical dendrite to capture voltage. To simulate STDP we adapted a previous protocol developed by Watanabe et al [14] that uses an *iClamp* class to introduce backpropagating action potentials to the soma while using the *Exp2Syn* class to simulate EPSP activity, and connecting these events with a *NetCon* connection.

### CA1 neuron model

The CA1 neuron model uses the previously published [15] rat hippocampus morphology available at NeuroMorpho [16] (ID: NMO_00227), and a modified version of the model described by Combe *et al*. 2018 [17]. The model is comprised of 194 sections and 728 segments that included: Ca^2+^ activated K^+^ channels, L-type Ca^2+^ channels, high threshold L-type Ca^2+^ channels, medium threshold R-type Ca^2+^ channels, T-type Ca^2+^ channels, N-type Ca^2+^ channels, fast-inactivating A-type channels (KA), slowly inactivating delayed-rectifier type (KDR) channels, Na^+^ channels (axon: nax.mod; soma/dendrite: na3.mod), Ca^2+^ pumps, persistent Na^+^ channels, R-type Ca^2+^ channels, non-inactivating voltage-dependent KM channels, and the Na^+^/Ca^2+^ exchanger.

The NCX current (*I*_*NCX*_) is specified by the product of an electrochemical factor (ΔE) and an allosteric factor (Allo) (Eqs 1,4–5) as described by Weber et al. [18] and was used to describe NCX-mediated Ca^2+^ and Na^+^ influx and efflux (Eqs 2–3):

$$I_{NCX}=(Allo)(\Delta E)$$ (1)

$$Calcium\;efflux=-J_{NCX}=-{\bar{I}}_{NCX}(Allo)(\Delta E)$$ (2)

$$Sodium\;efflux=+3J_{NCX}=+3({\bar{I}}_{NCX})(Allo)(\Delta E)$$ (3)

$$Allo=\frac{1}{1+(\frac{K_{mCaact}}{{\left[Ca\right]}_{i}}){}^{n}Hill}$$ (4)

$$\Delta E=V_{max}\left(\frac{{\left[{Na}^{+}\right]}_{i}^{3}{\left[{Ca}^{2+}\right]}_{o}e^{\frac{\gamma V_{m}F}{RT}}-{\left[{Na}^{+}\right]}_{o}^{3}{\left[{Ca}^{2+}\right]}_{i}e^{\frac{(\gamma -1)V_{m}F}{RT}}}{\left(k_{m(Na)}^{3}+{\left[{Na}^{+}\right]}_{o}^{3}{{)(k}_{m(Ca)}+{\left[{Ca}^{2+}\right]}_{o})(1+k}_{sat}e^{\frac{(\gamma -1)V_{m}F}{RT}}\right)}\right)$$ (5)

where, *V*_*max*_ is the maximal current, γ is the voltage dependence parameter, *k*_*sat*_ is the saturation factor, *k*_*m(Na)*_ and *k*_*m(Ca)*_ are the half saturation constants for [Na^+^]_o_ and [Ca^2+^]_o_ respectively, *F* is Faraday’s constant, *T* is temperature (in *Kelvin*), and *R* is the molar gas constant. Intracellular calcium and sodium concentrations were 50nM and 10mM respectively. Extracellular calcium and sodium concentrations are 2mM and 140mM respectively. See Weber et al. [18] for a complete description of the NCX model. NCX density was altered by changing *V*_*max*_ and allosteric factor altered by changing Allo based on previously reported data from Weber et al [18].

The Ca^2+^-dependent K^+^ current (*I*_*K[Ca]*_) is adapted from Destexhe et al. [19] (Eqs 6–7) and described as follows:

$$I_{K[Ca]}={\bar{g}}_{K[Ca]}m^{2}(V-E_{K})$$ (6)

$$m=-\frac{1}{\tau ({\left[Ca\right]}_{i})}[m-m_{\infty }({\left[Ca\right]}_{i})]$$ (7)

where ${\bar{g}}_{K[Ca]}$ = mho/cm^2^ represents the maximal permeability, *E*_*K*_ is the reversal potential, *m* is the activation variable of *I*_*K[Ca]*_, and [Ca]_i_ is the intracellular calcium concentration. The Ca^2+^-dependent functions are described (Eqs 8–9) as follows:

$$m_{\infty }({\left[Ca\right]}_{i})=\frac{\alpha {\left[Ca\right]}_{i}^{n}}{\alpha {\left[Ca\right]}_{i}^{n}+\beta }$$ (8)

$$\tau_{m}({\left[Ca\right]}_{i})=\frac{1}{\alpha {\left[Ca\right]}_{i}^{n}+\beta }$$ (9)

where *n* = 2, α and β are determined based on voltage and calcium concentration.

### Data representation and statistical analysis

Data were processed using *numpy* (ver. 1.16.4) and *pandas* (ver. 0.23.4). Plots were generated using *matplotlib* (ver. 2.2.3), and heatmaps were rendered using *PySpike* (ver. 0.6). Spike Sync [20,21] heatmaps were calculated and represented using the *spike_sync_matrix* function from PySpike [22], which computes the overall spike-synchronization value from pairs of spike-trains. Scatter plot data is represented as means ± standard deviation. The Mann Whitney U rank test from *SciPy stats* (ver. 1.3.0) was used to test array like samples where p = 0.05. The Tukey HSD test from the *StatsModels* Python module (ver. 0.9.0) was used to compare all pairwise comparisons with TukeyHSD confidence intervals at α = 0.05.

## Results

### Simulation model of CA1 pyramidal neuron simulates biologically realistic activity

**Fig 1:**
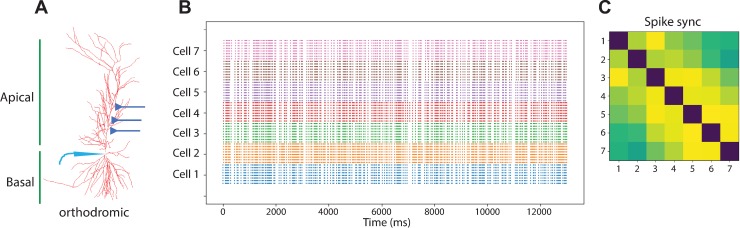
Gamma frequency stimulation of multi-compartmental CA1 model. **(A)** CA1 model uses the morphology data from rat hippocampus [15]. The *swc* file is accessible from NeuroMorpho [16] using the identifier NMO_00227. **(B)** Raster plot showing spike response patterns from seven different pyramidal cells recorded from the soma in response to 40Hz stimulation. Ten glutamatergic synapses are randomly positioned along the apical primary dendrite at least 100μm from the soma and the fractional randomness noise parameter set to one. **(C)** Spike sync heatmap reveals the overall spike-synchronization value from pairs of spike-trains. Each cell from **(B)** is represented on the X and Y-axes in **(C)**.

Using a morphologically realistic model from a CA1 pyramidal cell (Fig 1A) we simulated temporally and spatially defined CA3 excitatory input onto the CA1 pyramidal cell by activating 10 excitatory synapses at 40Hz gamma frequency in seven different CA1 pyramidal neurons (Fig 1B). Each of the 10 synapses were randomly positioned along the primary apical dendrite at least 100μm from the soma. Recordings were made at the soma and the resulting activity recorded as a spike if the threshold passed above 10mV (Fig 1B). Spike synchronization values from pairs of spike trains were calculated and plotted as heatmaps (Fig 1C) from data collected for each of the seven CA1 neurons used in Fig 1B. These data demonstrated unique patterns of activation for each cell. Based on 21 possible pairwise combinations for inter spike intervals (ISI) recorded for each neuron, the minimum similarity ≈ 1% and maximum similarity ≈ 27% (Python *difflib* module); mean firing rate (MFR, spikes per second) for each cell was: cell 1 = 15 s^-1^; cell 2 = 19.3 s^-1^; cell 3 = 15.2 s^-1^; cell 4 = 15.9 s^-1^; cell 5 = 14.6 s^-1^; cell 6 = 14.4 s^-1^; and cell 7 = 15.07 s^-1^. Taken together, we show that our model can simulate realistic activation patterns in response to gamma frequency stimulation.

### Na^+^/Ca^2+^ exchange current localization does not alter orthodromic CA1 activity

**Fig 2:**
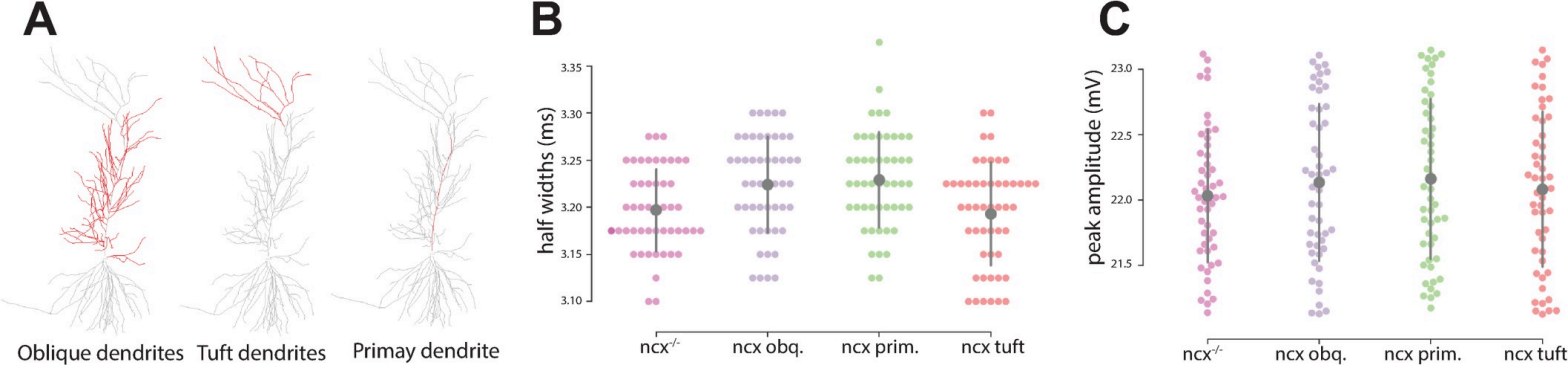
Simulation data of the sodium calcium exchange current during orthodromic propagation. **(A)** The CA1 model geometry was divided into regions based on dendritic morphology that included the oblique, tuft, and primary dendrite. **(B)** Half-width data was examined during forward propagation as a function of Na^+^/Ca^2+^ exchange current localization. (**C**) Peak voltage from the soma was recorded during forward propagation when Na^+^/Ca^2+^ exchange current was localized to specific sub-regions of CA1.

In order to understand the role of the Na^+^/Ca^2+^ exchange current in dendritic compartments, during forward signaling, we programmatically divided the dendrite into oblique, tuft, and primary regions (Figs 1A and 2A), and examined the effect of NCX localization on spike frequency. Recording from the soma of our model CA1 cell, we simulated Na^+^/Ca^2+^ exchange current activity using the CA1 model described above (Fig 1A–1C) in response to 40Hz gamma frequency stimulation and compared the output with simulations in which the Na^+^/Ca^2+^ exchange current was localized only to specific sub-regions of CA. We simulated activity in a total of 50 CA1 neurons after and used half-width time (Fig 2B) and spike amplitude (Fig 2C) as readouts to examine the effect of NCX localization. Our simulations included the following categories: 1) ncx-/-–Na^+^/Ca^2+^ exchange current model removed from the entire cell; 2) ncx oblique–Na^+^/Ca^2+^ exchange current model localized to the oblique dendrite; 3) ncx primary–Na^+^/Ca^2+^ exchange current model localized to the primary dendrite; and 4) ncx tuft–Na^+^/Ca^2+^ exchange current localized to the dendrite tuft. From these data we observed no significant difference in spike-width based on NCX localization using both Mann Whitney U (α = 0.05) and Tukey HSD (FWER = 0.05) tests (Fig 2B). Furthermore, by examining spike amplitude data as a function of NCX localization, we again did not observe significant differences (Fig 2C, Mann Whitney U (α = 0.05) and Tukey HSD (FWER = 0.05) tests). These findings were not surprising as the timescale of NCX activation would be too slow to meaningfully contribute to action potential dynamics and therefore leant further validation to our model’s morphological (Fig 1) and mathematical constraints (Fig 2).

### Presence of Na^+^/Ca^2+^ exchange current in oblique dendrites increases their excitability

**Fig 3:**
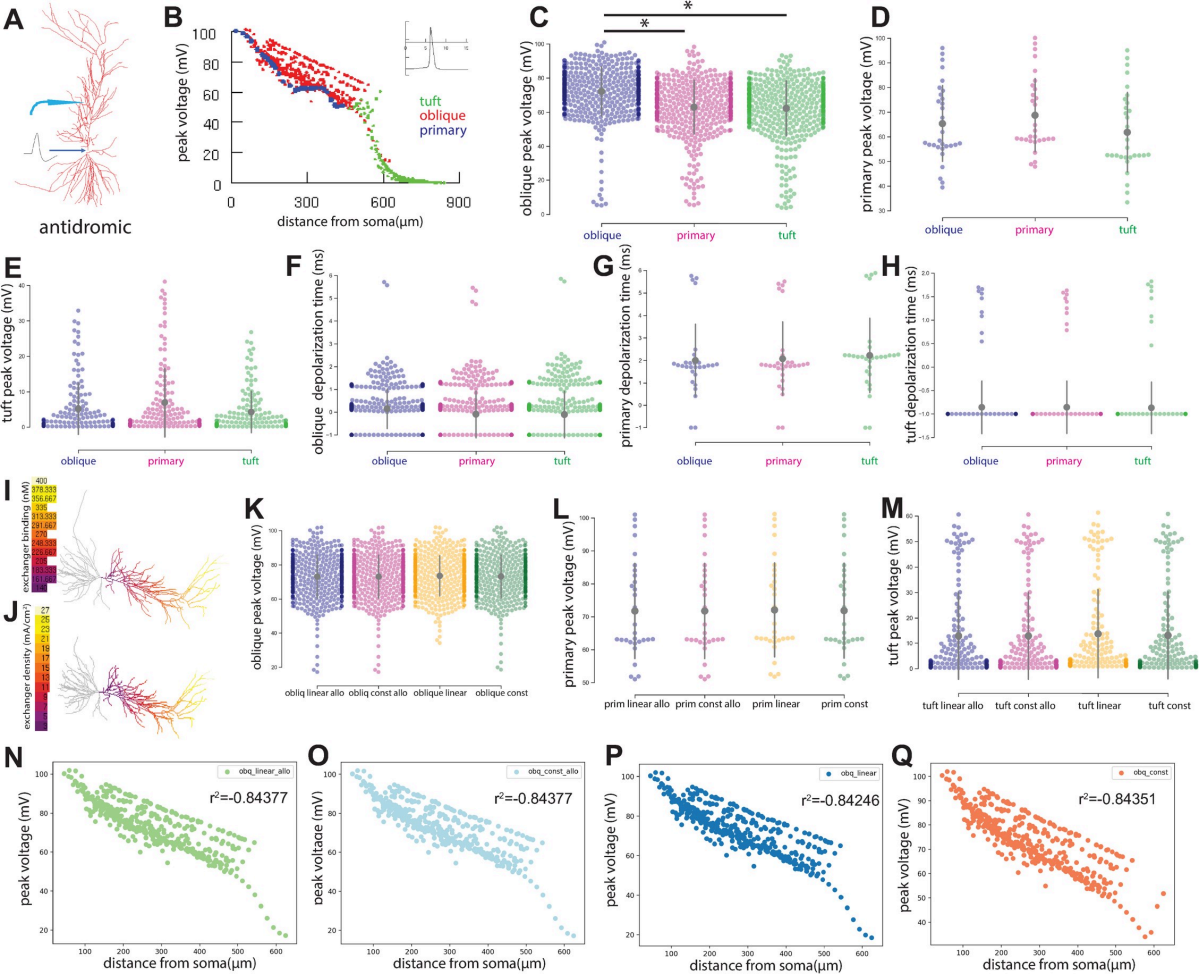
Simulation models on the effect of sodium calcium exchange current on back propagating action potentials. **(A)** Antidromic propagation was measured in all apical compartments after somatic stimulation. **(B)** Visual representation of the experimental set-up whereby absolute peak voltage for each apical compartment is represented by a colored dot and coded to map onto the primary, oblique, and tuft dendrite. Inset graphs the action potential triggered at the soma. (**C-E**) Absolute peak voltage from apical compartments was recorded during back propagating actin potentials while the Na^+^/Ca^2+^ exchange current was introduced to the oblique (**C**), primary (**D**), or tuft regions (**E**). **(F-H)** The depolarization time from each apical compartment was examined during back propagation when the Na^+^/Ca^2+^ exchange current was localized to the oblique (**F**), primary (**G**), or tuft dendrites (**H**). **(I-J)** Visual depictions of the linear models of NCX allostery **(I)** and NCX density **(J). (K-M)** Absolute peak voltage from oblique **(K),** primary **(L),** and tuft **(M)** apical regions were recorded while modeling linear and constant models of NCX allostery (*linear allo* and *const allo*), and linear and constant models of NCX density (*linear* and *const*). **(N-Q)** Correlation plots of absolute peak voltage from the oblique dendrite and distance from soma for the linear allostery model **(N),** constant allostery model **(O),** linear density model **(P),** and constant density model **(Q).** * indicates p < 0.005. Statistics calculated using the Mann Whitney U (α = 0.05) and Tukey HSD (FWER = 0.05) tests.

To study the effect of the Na^+^/Ca^2+^ exchange current on back propagating action potentials in CA1, we simulated a depolarizing current to the soma and recorded the resulting voltage change throughout the apical dendrite tree (Fig 3A). A representative dataset is illustrated in Fig 3B, whereby the absolute peak voltage for each apical compartment is represented by a single dot and color coded to map onto the primary apical dendrite, the oblique dendrite, and the tuft dendrite; the inset shows the action potential triggered at the soma. First, we examined the effect of Na^+^/Ca^2+^ exchange current localization in the apical tuft, oblique, or primary (trunk) on peak voltage in the oblique region (Fig 3C), primary dendrite (Fig 3D), and tuft region (Fig 3E). From this analysis we observed significant differences in the oblique peak amplitudes between the Na^+^/Ca^2+^ exchange current localized to the oblique and primary regions as well as oblique and tuft regions (p < 0.005). Next, we performed a similar set of experiments but this time instead of recording the absolute peak amplitude, we recorded the depolarization time (ms) for each compartment within the oblique (Fig 3F), primary (Fig 3G), and tuft dendritic regions (Fig 3H) when the Na^+^/Ca^2+^ exchange current was localized to the oblique, primary or tuft regions. In this case, we did not observe any significant differences in depolarization times. So far, our data suggested that the oblique dendrites were more excitable during back propagating action potentials in the presence of the Na^+^/Ca^2+^ exchange current in contrast to the other apical dendrite regions. Next, we sought to explore the effect of exchanger allostery and density on apical activity during back propagating action potentials. We devised gradient models of allostery and density based on the experimental work described by Weber et al. [18]. The model of NCX that we have been using thus far is a constant model (termed const) in that the density and allosteric constraints of the Na^+^/Ca^2+^ exchange current is uniform, we examined the effect of a linear model whereby the allostery increases linearly from the soma throughout the apical region (termed linear_allo), and implemented a similar linear model for Na^+^/Ca^2+^ exchange current density also (termed linear). Visual depictions of these linear models are shown in Fig 3I and 3J. To examine the effects of allostery and density on antidromic propagation, we recorded the peak absolute amplitudes from the oblique dendrites (Fig 3K), primary dendrites (Fig 3L), and tuft dendrites (Fig 3M) after implemented the linear allosteric model (linear allo), the constant allosteric model (const allo), the linear density model (linear) or the constant density model (const). From these analyses we did not observe any differences in peak voltage as a function of allosteric or density models (Fig 3K–3M). We also examined the depolarization times (ms) from oblique, primary, and tuft dendrites after implementing the allosteric and density models and also observed no significant differences (S1 Fig). When we plot the peak voltage against the soma distance for each allosteric model (Fig 3N and 3O) and density model (Fig 3P and 3Q), we can clearly see the almost identical correlation pattern in the case of the density models and identical patterns in the case of the allosteric models.

### NCX localization at the postsynaptic site is required to normalize spike-timing dependent plasticity

**Fig 4:**
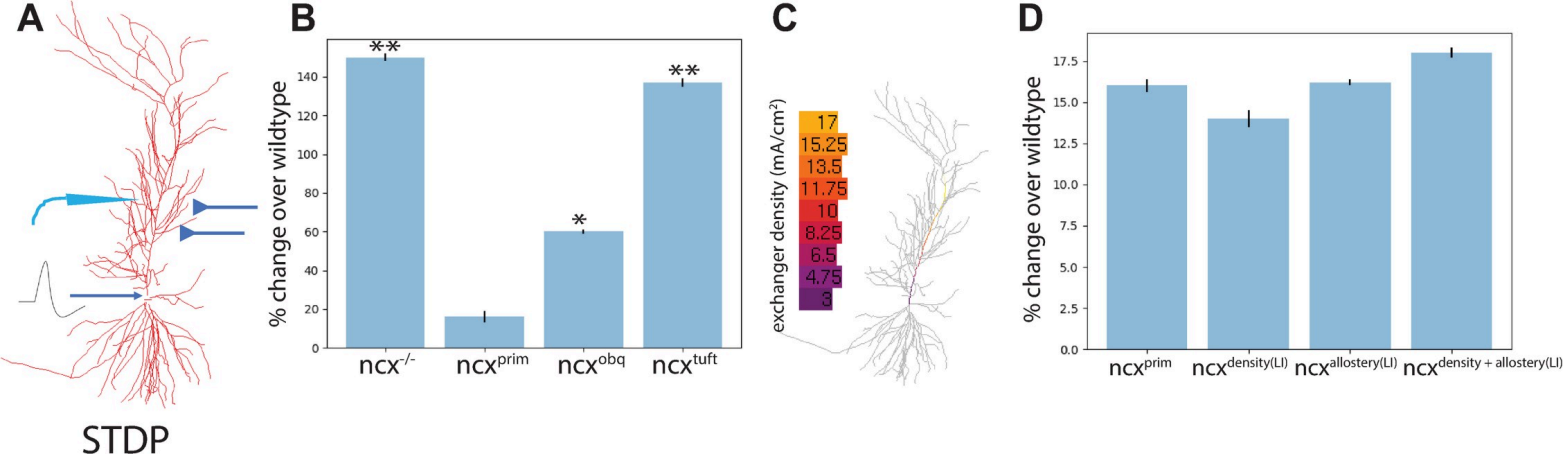
Removal of the Na^+^/Ca^2+^ exchange current increases potentiation. (**A**) STDP paradigm described previously [14] was used to pair back propagating action potentials with EPSPs to simulate potentiation at the synapse. (**B**) Potentiation of the synapse was examined in the absence of the Na^+^/Ca^2+^ exchange current (ncx^-/-^), when the Na^+^/Ca^2+^ exchange current is localized only to the primary dendrite (ncx^prim^), when the Na^+^/Ca^2+^ exchange current is localized the oblique dendrite region (ncx^obq^), or when the Na^+^/Ca^2+^ exchange current was localized to the tuft dendrite region (ncx^tuft^). **(C)** Graphical heatmap representation of the linear increase of the Na^+^/Ca^2+^ exchange current along the primary dendrite. **(D)** Comparison of STDP responses from the Na^+^/Ca^2+^ exchange current localized only to the primary dendrite (ncx^prim^) against linear models of NCX density (ncx^density(LI)^), allostery (ncx^allostery(LI)^), and density plus allostery (ncx^density+allostery(LI)^) along the primary dendrite. ** indicates p < 0.005 * indicates p < 0.05.

Next, we employed a STDP paradigm developed by Watanabe et al. [14] to simulate potentiation in response to a back-propagating action potential alongside EPSPs (Fig 4A). The EPSPs were induced at the distal portion of the primary dendrite. The pairing of the antidromic action potential and the EPSPs result in potentiation at the synapse when paired within 35ms, as compared with unpaired stimuli. Here we set out to examine whether the Na^+^/Ca^2+^ exchange current may contribute to this potentiation. For this analysis we compared the potentiation response across four different scenarios: 1) when NCX was removed (ncx^-/-^); 2) when NCX localized only to the primary dendrite (ncx^prim^); 3) when NCX localized only to the oblique localization (ncx^obq^); and 4) when NCX localized only to the tuft dendrite (ncx^tuft^). From these simulations we observed a significantly increased potentiation response when the Na^+^/Ca^2+^ exchange current was removed as compared with wildtype (Fig 4B, ncx^-/-^ versus wt: p< 0.005). A similar increase in potentiation was observed when NCX was localized to the apical tuft dendrite and to a lesser extent when NCX was localized to the apical oblique dendrite regions (Fig 4B, ncx^tuft^ versus wt: p < 0.005; ncx^obq^ versus wt: p < 0.05). Interestingly, when NCX was localized to the primary dendrite region, which is also the site of synaptic input, the potentiation was reduced to near normal levels (Fig 4B, ncx^prim^ versus wt: p > 0.05). Inward sodium current during back propagating action potentials triggered reverse mode NCX activity (S2 Fig). These data suggest that removal of the Na^+^/Ca^2+^ exchange current increase the STDP response, and that localization of the Na^+^/Ca^2+^ exchange current to the postsynaptic site is required for normal responses. Next, we sought to investigate a possible role for NCX allostery and density along the primary dendrite in normalizing STDP. Similar to the linear increase model of allostery and density described above (see Fig 3I and 3J), we implemented a linear increasing model of NCX allostery and density along the primary dendrite to examine how allosteric regulation and density may alter STDP. Graphical illustration of the density linear model is shown in Fig 4C. A similar model of allostery was also developed (graphical illustration not shown). Next, we recorded the STDP response in four different test cases: 1) the Na^+^/Ca^2+^ exchange current was localized to the primary dendrite (ncx^prim^); 2) Na^+^/Ca^2+^ exchange current linear density model in the primary dendrite (ncx^density(LI)^); 3) Na^+^/Ca^2+^ exchange current linear allostery model in the primary dendrite (ncx^allostery(LI)^); and 4) Na^+^/Ca^2+^ exchange current linear density and linear allosteric models in the primary dendrite (ncx^density+allostery (LI)^). No significant differences were observed in the potentiation responses across these four scenarios, suggesting that, at least in our simulations, the density and allostery does not scale with STDP responses (Fig 4D). Taken together, our data describes a role for the sodium calcium exchange current in normalizing STDP responses. This observation is in keeping with previous data which has found that genetically removing NCX can result in increased synaptic plasticity [6,8].

### Opposing activity between NCX and the Ca^2+^-dependent K^+^ current determines spike-timing dependent synaptic plasticity levels

**Fig 5:**
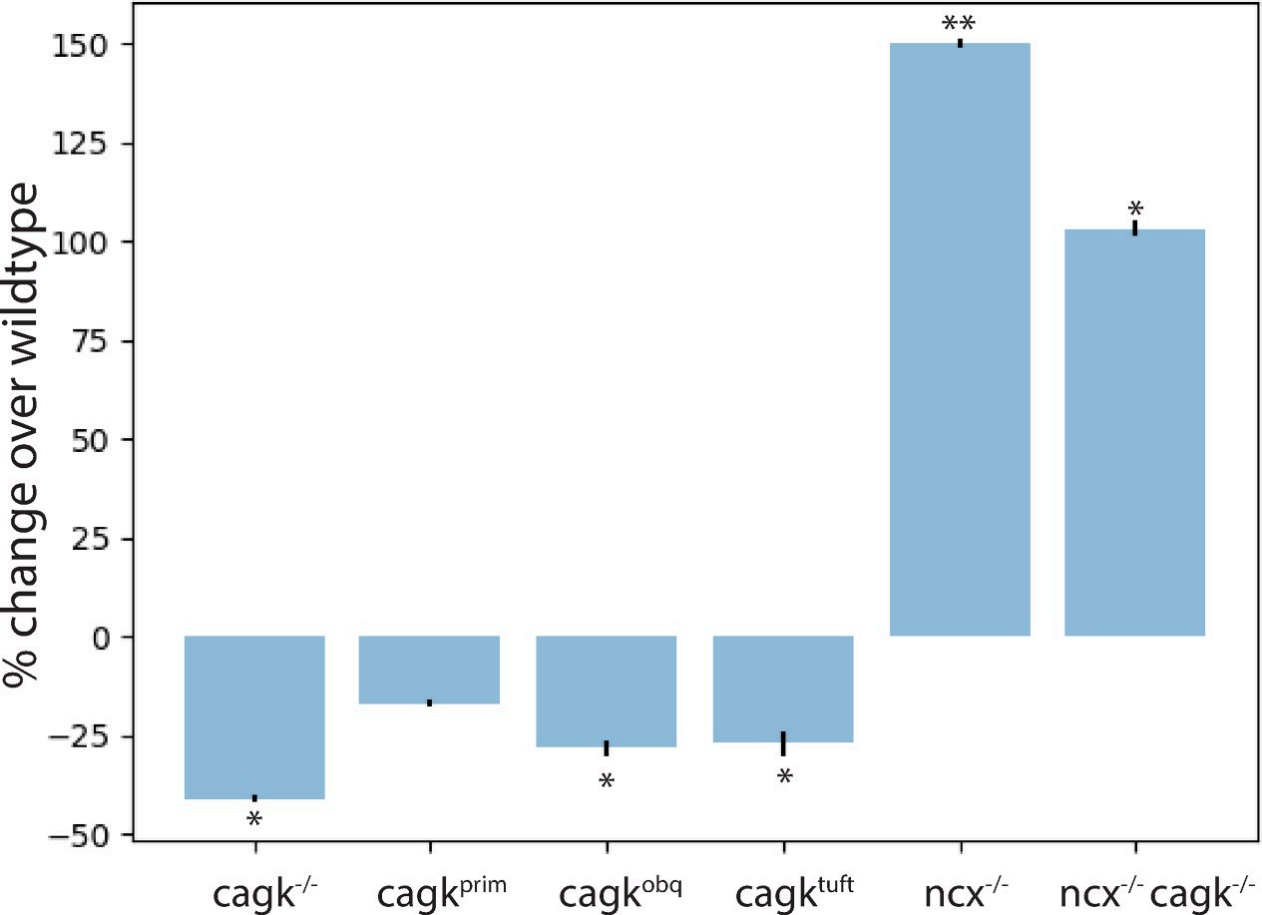
The Ca^2+^ activated K^+^ current is required for STDP. Comparison of STDP responses after Ca^2+^ sensitive K^+^ current mechanism removal (cagk^-/-^), insertion of the Ca^2+^ sensitive K^+^ current into the primary dendrite region (cagk^prim^), insertion of the Ca^2+^ sensitive K^+^ current into the oblique dendrite region (cagk^obq^), insertion of the Ca^2+^ sensitive K^+^ current into the tuft dendrite region (cagk^tuft^), and removal of both the Na^+^/Ca^2+^ exchange current and the Ca^2+^ sensitive K^+^ current from the model (cagk^-/-^; ncx^-/-^) as well as removal of the Na^+^/Ca^2+^ exchange current (ncx^-/-^) for comparison. ** indicates p < 0.005, * indicates p < 0.05.

To gain more mechanistic insight into how the Na^+^/Ca^2+^ exchange current influences STDP, we screened other mechanisms in our model for a role in STDP. From this screen, we identified the Ca^2+^ sensitive K^+^ channel mechanism as an important regulator of STDP. We found that removal of this mechanism (cagk^-/-^) significantly reduced the potentiation response (Fig 5, cagk^-/-^ versus wt: p < 0.05). By inserting the Ca^2+^ sensitive K^+^ current into the primary dendrite region, normal potentiation was restored (Fig 5, cagk^-/-^ versus cagk^prim^, p < 0.05). Inserting the Ca^2+^ sensitive K^+^ current into the oblique or tuft dendrite regions did not increase potentiation to the same level as when inserted into the primary dendrite region (Fig 5, cagk^obq^ or cagk^tuft^ versus cagk^prim^, p < 0.05). This suggests that similar to the Na^+^/Ca^2+^ exchange current, the Ca^2+^ sensitive K^+^ current likely functions primarily at the postsynaptic site to influence STDP responses. To explore the relationship between the Na^+^/Ca^2+^ exchange current and Ca^2+^ sensitive K^+^ current during STDP, we examine the double mechanism knockout (cagk^-/-^; ncx^-/-^) which removed both the Na^+^/Ca^2+^ exchange current and the Ca^2+^ sensitive K^+^ current from the model. In this case we observed reduced potentiation as compared to similar to ncx^-/-^ alone (Fig 5, cagk^-/-^; ncx^-/-^ versus ncx^-/-^ p > 0.05). We also examined K^+^ currents (*I*_*K+*_) during bAPs and found that the inward *I*_*Na+*_ during bAPs led to greater activation of Ca^2+^ sensitive K^+^ current channels from reverse mode NCX that increases outward *I*_*K+*_. Removing the Na^+^/Ca^2+^ exchange mechanism thereby not only reduces inward *I*_*Ca2+*_ but also abrogates the Ca^2+^ sensitive K^+^ current by reducing outward *I*_*K+*_: without the ncx^-/-^ mechanism, the outward *I*_*K+*_ was reduced by an average of 23.3% for peak 1 and 37.5% for peak 2 as compared with wildtype. Taken together, our results reveal that the Ca^2+^ sensitive K^+^ current is required at the postsynaptic site to promote STDP while the Na^+^/Ca^2+^ exchange current is required at the postsynaptic site to normalize STDP responses, which suggest that together they create a negative feedback mechanism to deliver an appropriate set-point for STDP responses.

## Discussion

The timescale of Ca^2+^ accumulation during synaptic signaling in the postsynaptic cell is critical in determining synaptic plasticity [23–25]. In CA1 neurons, action potentials move into proximal dendrites and open voltage sensitive Ca^2+^ channels that result in near global Ca^2+^ elevations in spines and dendrites where NCX and plasma membrane Ca^2+^ pumps represent the major clearance mechanisms [26]. Given the central role for NCX in Ca^2+^ homeostasis, it denotes a unique substrate for tuning Ca^2+^ levels, and in turn synaptic plasticity. Previous work has shown that forward mode NCX functions at the postsynaptic side to promote Ca^2+^ clearance during depolarization at synaptic terminals [6]. Furthermore, the presence of NCX at the synapse makes it a unique target for depolarizing currents from back propagating action potentials (bAPs) and STDP. By developing multi-compartment models of NCX function in pyramidal cells, we have been able to make several interesting observations. Our simulation data suggest that NCX plays an important role in STDP by adjusting residual Ca^2+^ levels from EPSP events; manipulating NCX localization allowed us to uncover a mechanism whereby deletion of NCX results in elevated potentiation during pairing of bAPs with EPSPs. This suggest that NCX functions to set an appropriate set-point for STDP, and that defects in this normalization mechanism results in altered STDP. Our simulation data also demonstrated a role for the Ca^2+^-activated K^+^ channel current in promoting potentiation during pairing of bAPs with EPSPs. Previous data from both cortical and hippocampal pyramidal neurons has shown that bAPs can activate small conductance Ca^2+^-activated K^+^ channels (SK channels), and thereby function to gate STDP during bAP pairing with EPSP [27]. A novel insight from our data suggests that both NCX and Ca^2+^-activated K^+^ channels may function in opposing ways in postsynaptic cells to adjust STDP during bAP pairing with EPSP. While limitations do exist within our simulation models, our findings are suggestive of an integrative mechanism by NCX and small conductance Ca^2+^-activated K^+^ channels to modulate synaptic plasticity.

## Supporting information

S1 Fig Simulation models on the effect of NCX allostery during back propagating action potentials (bAPs). Depolarization times were recorded during back propagation when the Na^+^/Ca^2+^ exchange current was localized to the oblique (**A**), tuft (**B**), or primary dendrites (**C**) while modeling linear and constant models of NCX allostery (*linear allo* and *const allo*), and linear and constant models of NCX density (*linear* and *const*).
(TIF)

S2 Fig Reverse mode NCX during paired pulse bAPs with EPSPs. Na^+^/Ca^2+^ exchange currents were examined during bAPs paired with EPSPs to examine the direction of Ca^2+^**(A)** and Na^+^ **(B)** ion exchange.
(TIF)

## Abbreviations

NCX: Sodium Calcium Exchanger
EPSP: Excitatory post synaptic potential
STDP: spike-timing dependent plasticity
