# Supplementary figures and images for: Simulation model of CA1 pyramidal neurons reveal opposing roles for the Na+/Ca2+ exchange current and Ca2+-activated K+ current during spike-timing dependent synaptic plasticity
Source: PLoS One. 2020 Mar 9;15(3):e0230327. doi: 10.1371/journal.pone.0230327 (PMC7062500; doi:10.1371/journal.pone.0230327)

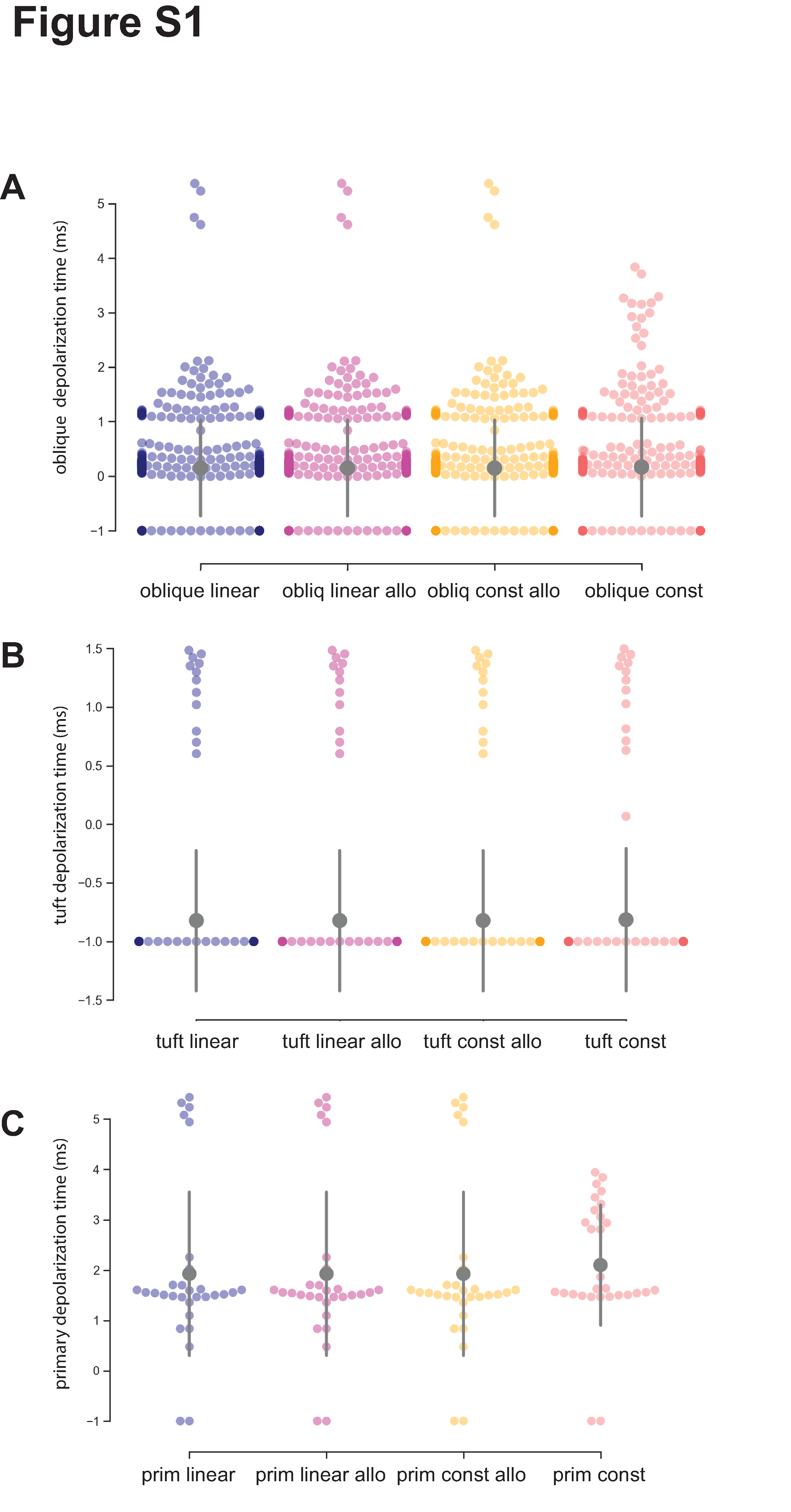

Supplement: S1 Fig — Depolarization times were recorded during back propagation when the Na+/Ca2+ exchange current was localized to the oblique (A), tuft (B), or primary dendrites (C) while modeling linear and constant models of NCX allostery (linear allo and const allo), and linear and constant models of NCX density (linear and const). (TIF) [file pone.0230327.s001.tif]

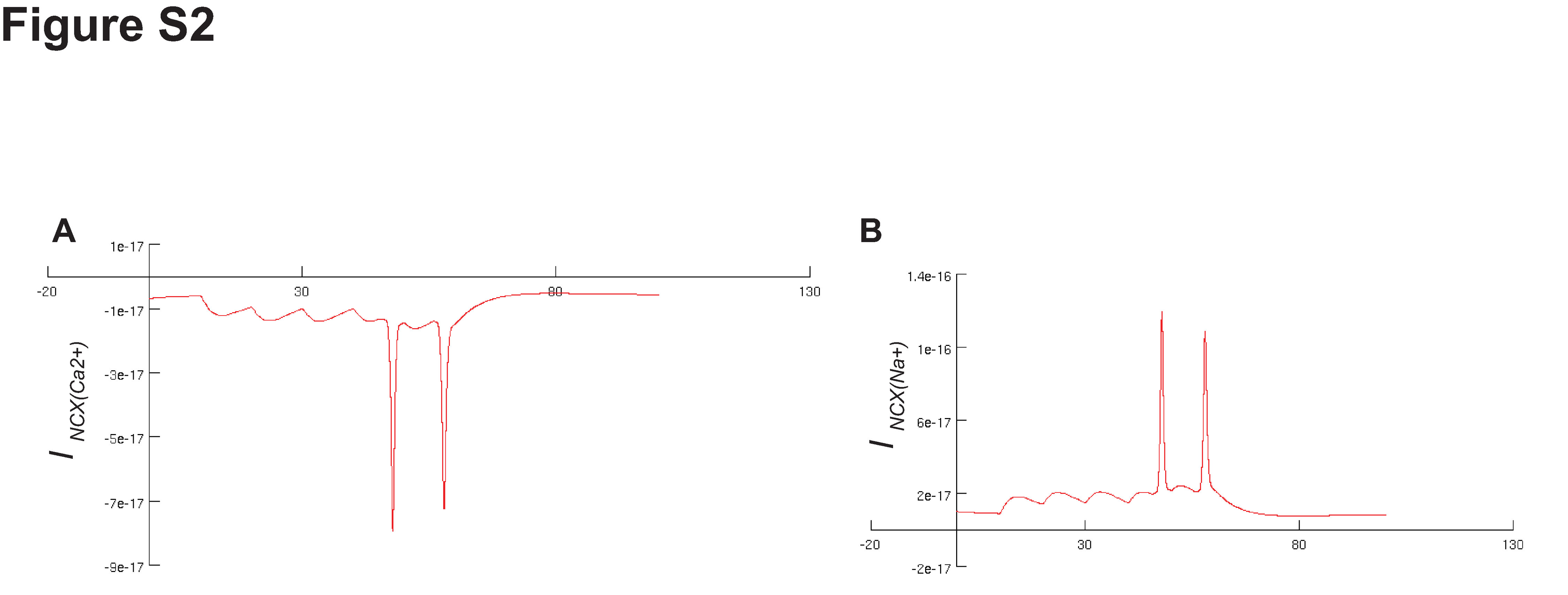

Supplement: S2 Fig — Na+/Ca2+ exchange currents were examined during bAPs paired with EPSPs to examine the direction of Ca2+(A) and Na+ (B) ion exchange. (TIF) [file pone.0230327.s002.tif]
